# Insertion in the N-Terminal Domain of the SARS-CoV-2 Spike Glycoprotein Affects Antibody Recognition and Phenotypic Properties

**DOI:** 10.3390/v18030277

**Published:** 2026-02-24

**Authors:** Elena A. Ermolaeva, Anna N. Zyrina, Dina I. Sirazova, Alexander S. Lunin, Anton S. Motov, Anastasia D. Chernavtseva, Olga S. Gancharova, Liubov I. Kozlovskaya, Anna A. Shishova, Alexandra A. Siniugina, Aydar A. Ishmukhametov

**Affiliations:** 1 Chumakov FSC R&D IBP RAS (Institute of Poliomyelitis), 108819 Moscow, Russia; 2 Institute for Translational Medicine and Biotechnology, First Moscow State Medical University (Sechenov University), 117418 Moscow, Russia; 3 Belozersky Institute of Physico-Chemical Biology, Lomonosov Moscow State University, 119991 Moscow, Russia

**Keywords:** SARS-CoV-2, Delta variant, glycoprotein spike, N-terminal domain, mutations, immune evasion, pathogenicity

## Abstract

SARS-CoV-2, which causes COVID-19, continues to circulate around the world, making it necessary to study the impact of rapidly emerging mutations on escape from neutralizing antibodies and pathogenesis. While RBD mutations are well characterized, mutations in the N-terminal domain (NTD) of the spike protein remain comparatively understudied despite their relevance to antibody recognition. This study investigates two phenotypically distinct SARS-CoV-2 mutants, which exhibited differences in plaque morphology on Vero cells. Whole-genome sequencing via Illumina identified a novel 12-nucleotide insertion in the spike NTD. This insertion induced a frameshift, introducing five new amino acids potentially altering viral behavior, receptor interactions, and antibody detection in ELISAs. The study further explores the pathogenicity of these variants in a hamster model. These findings underscore the importance of monitoring NTD mutations, which may contribute to immune evasion and influence therapeutic antibody efficacy, highlighting gaps in current research on SARS-CoV-2 evolution.

## 1. Introduction

Coronaviruses are a large group of viruses capable of infecting various mammalian species, causing both mild and severe human respiratory disease [1].

SARS-CoV-2 belongs to the genus *Betacoronavirus*, subfamily *Orthocoronavirinae*, family *Coronaviridae* [2]. The virus is enveloped, with three S glycoproteins forming a spike on the surface of the virus, which mediates attachment to the cell and fusion of the viral envelope with the cell membrane. Protein S is a precursor protein and after sorption on angiotensin-converting enzyme 2 (ACE2) is cleaved by a furin-like protease into receptor-binding subunit S1 and fusion subunit S2 [3,4,5]. Numerous non-synonymous substitutions, especially in the spike-encoding region, led to the formation of new SARS-CoV-2 variants with altered phenotypic properties [6].

To date, five SARS-CoV-2 variants have been designated as Variants of Concern (VOCs) by the World Health Organization: Alpha (PANGO lineage B.1.1.7), Beta (B.1.351), Gamma (P.1), Delta (B.1.617.2/AY sublines) and Omicron (B.1.1.529 and its BA sublineages, including BA.1, BA.2, and BA.5) [6]. During 2020–2021, Alpha and Beta VOCs were dominant in SARS-CoV-2 circulation. The situation changed in December 2021, when the Omicron variant caused an increase in COVID-19 incidence [7].

The Delta variant (B.1.617.2) was first discovered in India in late 2020 [8]. It has a unique set of mutations, nine of which are located in the spike protein (T19R, G142D, 156-157del, R158G, L452R, T478K, D614G, P681R, D950N) [9]. Mutations in spike protein resulted in enhanced binding affinity to the cell’s ACE2 receptor, which increased Delta variant transmissibility [10]. The mutations also made the Delta variant resistant to some monoclonal antibodies, such as bamlanivimab, and resulted in decreased efficacy of sera from COVID-19 convalescents [11].

The decreasing efficacy of already developed neutralizing monoclonal antibodies made it necessary to study mutations in the genomes of various SARS-CoV-2 variants [12]. Most of the studies devoted to antibodies are focused on the RBD. However, analyses of the immunoglobulin G (IgG) antibody repertoire in the sera of convalescent COVID-19 patients showed that a substantial proportion of neutralizing antibodies bind to epitopes outside the RBD [13], almost half of the neutralizing antibodies were found to bind the NTD [14]. An NTD-specific monoclonal antibody (COV1-65) isolated from a COVID-19 patient effectively neutralized the virus in vitro [15]. Moreover, viruses with ΔH69/ΔV70 deletion in NTD showed increased infectivity and escaped from neutralizing antibodies in convalescent plasma [16]. In addition, the presence of Y144del and 242-244del mutations in NTD confers resistance to human neutralizing monoclonal antibodies [17]. Consequently, an increasing number of studies are now focused on the impact of NTD mutations on the immunogenicity, pathogenesis and immune-evasion capacity of SARS-CoV-2 [18,19,20,21,22,23].

Given that mutations in the NTD can alter viral antigenicity and potentially affect virulence, it is important to evaluate their biological impact in vivo. Small animal models such as ferrets, mice expressing human ACE2, and Syrian hamsters have been used to study the immune response and pathogenesis. These models reproduce key features of human disease, including a robust cytokine burst and early infiltration of innate and adaptive immune cells [24,25]. The Syrian hamster model was used to study the pathogenesis of the Delta variant with detailed descriptions of the disease course, viral load in nasal washes and lung tissue, and body weight dynamics in animals infected with different Delta sublineages [26]. As in humans, the Delta variant causes more severe disease in the Syrian hamster compared to previous coronavirus variants, justifying the use of the model to study pathogenesis [27]. The Syrian hamster model can also be used to study biomarkers and indicators of COVID-19, which will further help in diagnosis and prevention [28].

The present study describes two phenotypically distinct variants of SARS-CoV-2, differing by a mutation in the region of the genome encoding the NTD of the spike protein, and examines their recognition by neutralizing anti-spike antibodies as well as their pathogenesis in the Syrian hamster model.

## 2. Materials and Methods

### 2.1. Viruses and Cells

The original (parent) Delta variant strain 4724d (GISAID EPI_ISL_8799478) SARS-CoV-2 was obtained from the collection of the FSASI “Chumakov FSC R&D IBP RAS” (Institute of poliomyelitis) (Moscow, Russia). Vero cells (RCB 10-87, WHO Biologicals, Geneva, Switzerland) were cultured in DMEM (FSASI “Chumakov FSC R&D IBP RAS” (Institute of poliomyelitis), Moscow, Russia) supplemented with 10% fetal bovine serum (Gibco, New York, NY, USA) and penicillin-streptomycin (Gibco, New York, NY, USA) maintained at 37 °C in a 5% CO_2_.

### 2.2. Virus Titration (TCID_50_) Assay in Vero Cells

Virus titration was performed in 96-well plates using Vero cell monolayers cultured for 72 h. Ten-fold serial dilutions of the virus-containing suspension were prepared in cell culture medium and used to inoculate the cells (100 µL per well, eight replicates per dilution). Following incubation at 37 °C in a humidified atmosphere containing 5% CO_2_, cytopathic effect (CPE) was monitored daily for 4 days. Viral titers were calculated using the Kärber method and expressed as the 50% tissue culture infectious dose per milliliter (TCID_50_/mL).

### 2.3. Plaque Assay

Serial 10-fold dilutions of the virus-containing suspension were prepared in DMEM, and 300 µL of each dilution was added to a well of a six-well cell culture plate (Eppendorf, Hamburg, Germany) containing a Vero cell monolayer grown for 72 h. After 30 min of virus adsorption at 37 °C in a 5% CO_2_ atmosphere, the cell monolayer was overlaid with 2 mL of chilled two-component overlay medium consisting of agar (final concentration 0.8%) and 2% fetal bovine serum (Gibco, New York, NY, USA) in Earle’s saline solution (FSASI “Chumakov FSC R&D IBP RAS” (Institute of poliomyelitis), Moscow, Russia) supplemented with 3% sodium bicarbonate and 0.5% antibiotic solution (Gibco, New York, NY, USA; 5000 Units/mL penicillin + 5000 µg/mL streptomycin) [22]. Plaques were counted and measured on day 5 post infection.

### 2.4. Sample Preparation for Sanger Sequencing

Viral RNA was isolated from the culture supernatant using the Extract RNA reagent (Eurogen, Moscow, Russia) followed by chloroform (Component-Reagent, Moscow, Russia) extraction. cDNA was synthesized from the isolated RNA using the reverse transcription with M-MuLV reverse transcriptase (New England Biolabs, Ipswich, United Kingdom). To amplify the genomic region encoding the spike (S) protein, the obtained cDNA was used as a template for PCR with primers COVID_21095_F (GAGGGTTTTTTCACTTACAT) and COVID_22118_R (GAAATTACCCTGTTTTCCTTC) using 5× ScreenMix-HS polymerase (Eurogen, Moscow, Russia). RT and PCR were performed according to the manufacturer’s instructions, with an annealing temperature of 55 °C. The obtained amplicons were purified from reaction mixture residues using the QIAquick Gel Extraction Kit (Qiagen, Hilden, Germany) according to the manufacturer’s instructions.

The reaction mixture for Sanger sequencing contained 20 ng of the obtained amplicons, primers used for PCR (3 pM/μL), and the mixture was brought to 13 μL with water. Sanger sequencing was performed on a sequencer Applied Biosystems 3130 Genetic Analyzer.

The received files in FASTA format were analyzed in the program Unipro UGENE. The SARS-CoV-2 genome (NC_045512.2; severe acute respiratory syndrome coronavirus 2 isolate Wuhan-Hu-1) was used as a reference.

### 2.5. Sample Preparation for NGS and Bioinformatics Processing

Viral RNA was isolated from the culture supernatant using the Extract RNA reagent (Eurogen, Moscow, Russia) followed by chloroform (Component-Reagent, Moscow, Russia) extraction. The obtained RNA was treated with DNase E (Eurogen, Moscow, Russia). Sequencing was performed by a third-party commercial organization. The quality of sequencing data was assessed using FastQC. Based on the FastQC report, adapter sequences (TruSeq), the first 15 nucleotides (parameter HEADCROP:15), and reads with a quality score below 30 (parameter TRAILING:30) were trimmed using Trimmomatic [29]. The filtered reads were then mapped to the SARS-CoV-2 reference genome (NC_045512.2; severe acute respiratory syndrome coronavirus 2 isolate Wuhan-Hu-1) using BWA with default parameters [30]. Variant calling was performed using the FreeBayes package [31] with the parameter—min-alternate-fraction 0.1 relative to the reference genome. The identified mutations were manually verified using IGV (Integrative Genomics Viewer, version 2.19.7).

### 2.6. Enzyme-Linked Immunosorbent Assay (ELISA)

Serial three-fold dilutions of the virus were prepared in phosphate-buffered saline (PBS; Merck, Sigma-Aldrich, Burlington, MA, USA) and transferred to 96-well clear flat-bottom high-binding polystyrene microplates (Corning, NY, USA). The plates were incubated overnight at 4 °C to allow virus adsorption. Wells were then filled with 200 µL of blocking solution (4% skimmed milk in PBS-T buffer containing 0.05% Tween-20) and incubated for 1 h at 37 °C. Primary antibodies were added and incubated for 1 h at 37 °C: a monoclonal antibody against the SARS-CoV-2 spike protein (2 mg/mL; 1:2500; FSASI “Chumakov FSC R&D IBP RAS” (Institute of poliomyelitis), Moscow, Russia), polyclonal antibody against SARS-CoV-2 spike protein (600-401-MS9, Rockland, NY, USA) and a polyclonal antibody against the N protein (1 mg/mL, 1:500; FSASI “Chumakov FSC R&D IBP RAS” (Institute of poliomyelitis), Moscow, Russia). Secondary antibodies were then applied and incubated for 1 h at 37 °C: HRP-conjugated anti-human antibodies (1:20,000; Promega, Madison, WI, USA) for spike detection and HRP-conjugated anti-rabbit antibodies (1:10,000; Promega, USA) for N protein detection. Finally, TMB liquid substrate solution for ELISA (Merck, Sigma-Aldrich, USA) was added to each well and incubated until color development and optical density was measured at 450 nm using a microplate reader (Multiscan, Thermo Fisher Scientific, Waltham, MA, USA).

### 2.7. Western-Blot

The inactivated virus-containing fluid was loaded onto a 12% SDS–polyacrylamide gel and viral proteins were separated by electrophoresis, followed by transfer to a nitrocellulose membrane. The membrane was blocked for 2 h with 5% skimmed milk prepared in TBS-T buffer (Tris-buffered saline containing 0.1% Tween-20). After blocking, the membrane was incubated for 1 h at room temperature with monoclonal antibodies against the SARS-CoV-2 spike protein (2 mg/mL; 1:2500; FSASI “Chumakov FSC R&D IBP RAS” (Institute of poliomyelitis), Moscow, Russia) and N-protein (1 mg/mL, 1:500; FSASI “Chumakov FSC R&D IBP RAS” (Institute of poliomyelitis), Moscow, Russia), followed by incubation for 1 h with HRP-conjugated anti-human secondary antibodies (1:20,000; Promega, USA). The membrane was then washed three times for 15 min each with TBS-T buffer. Protein bands were visualized using an ECL detection kit (Bio-Rad, Hercules, CA, USA) and imaged with the GeneSys gel documentation system (GeneSys, Menlo Park, CA, USA).

### 2.8. Infection of Syrian Hamsters

Outbred Syrian hamsters, females, 90–100 g (8 weeks old) were purchased from Scientific Center of Biomedical Technologies, branch Stolbovaya, Russia. Animals were randomly distributed between three groups: intact (N = 6), Delta l.p. (N = 15), and Delta s.p. (N = 15). Experimental hamsters were infected intranasally with 10^4^ TCID50 (25 µL into each nostril) of each cloned virus. Animals were observed and weighed daily and oropharyngeal swabs were collected. Five animals from each infected group were euthanized on days 3, 7 and 10 post infection (d p.i.). Lungs were collected for virological and histological investigation. Left lungs were homogenized in physiological saline using TissueLyser (Qiagen, Hilden, Germany). Swabs and organ suspensions were examined for the presence of viral RNA by 1-step RealTime RT-PCR kit (POLYVIR SARS-CoV-2, Lytech, Moscow, Russia) according to the manufacturer’s protocol.

### 2.9. Histology

Lung samples of 3 experimental groups (left lung of each experimental animal) were fixed in 10% neutral buffered formalin for 48 h at room temperature, dehydrated in isopropyl alcohol and embedded in paraffin medium. Formalin-fixed paraffin embedded tissue blocks were used to obtain two micrometer-thick cross-sections from all lung lobes. All obtained representative sections were stained with Mayer’s hematoxylin (Biovitrum, St. Petersburg, Russia) and eosin (Biovitrum, Russia) using Leica ST5010 Autostainer XL (Leica, Wetzlar, Germany), and 3 lungs from each experimental group (9 lungs in total) were stained by Van Gieson trichrome stain (Biovitrum, Russia) to visualize collagen. Histological preparations were converted into digital format using a Aperio AT2 (Leica, Germany) brightfield Digital Pathology scanner. The resulting digital slides of histological preparations were examined by a certified pathologist using Leica Aperio Imagescope software (Aperio ImageScope 12.4.6). Representative microphotographs were obtained using the snapshot tool in the indicated software. The severity of the pathological process in the lungs of each animal was assessed according to the pneumonia intensity score (0–4), where: 0—pathological processes (pneumonia) are not visible, 1—mildly pronounced, 2—moderately pronounced, 3—sharply pronounced, 4—extremely pronounced. The area of the lesions was also visually assessed according to area score (0–4), where 0—no changes or the lesion involves less than 10% of the lung section area; 1—lesion involves 10–25% of the area; 2—lesion involves 25–50% of the area; 3—lesion involves 50–75% of the area; 4—lesion involves 75–100% of the lung section area.

### 2.10. Ethical Statement

The animal study protocol was approved by the Ethics Committee of FSBSI “Chumakov FSC R&D IBP RAS” (№191023-2, date of approval 19 October 2023).

## 3. Results

### 3.1. Two Variants of Delta Variant of SARS-CoV-2 with Different Plaque Phenotypes in Vero Cells

**Figure 1:**
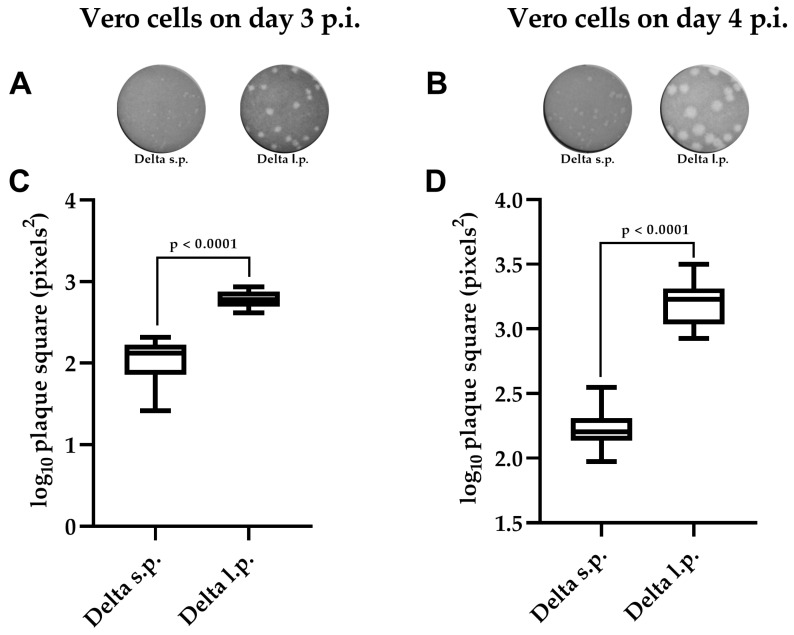
Plaque phenotypes of Delta s.p. (small plaques) and Delta l.p. (large plaques). (**A**) Plaque phenotype of the isolated viruses under agar overlay in Vero cells on day 3 p.i.; (**B**) plaque phenotype of the isolated viruses under agar overlay in Vero cells on day 4 p.i.; (**C**) the difference in the logarithmic areas of plaques formed by the obtained viruses on day 3 p.i.; (**D**) the difference in logarithmic areas of plaques formed by the obtained viruses on day 4 p.i.. The images were analyzed in ImageJ/Fiji (Java 1.8.0_45). All data are presented as mean with standard deviation. The *p*-value was obtained by Mann–Whitney test using GraphPad Prism 8.2.1.

Two phenotypically distinct variants of SARS-CoV-2, designated Delta s.p. (small plaques) and Delta l.p. (large plaques), were plaque-cloned from 7th passage of a patient-derived Delta variant isolate in Vero cells. To obtain phenotypically homogeneous viral populations, plaque cloning was subsequently performed three times. After these three passages, phenotypically homogeneous clones of the Delta SARS-CoV-2 were obtained (Figure 1).

After obtaining phenotypically homogeneous variants, we compared plaque areas produced by the Delta s.p. and Delta l.p. viruses on days three and four post infection (d p.i.). Representative plaque phenotype images are shown for day three (A) and day four (B) (Figure 1). Quantitative analysis demonstrated that the Delta l.p. variant formed significantly larger plaques than the Delta s.p. variant on both days (Figure 1C,D). The differences in log10-transformed plaque areas were statistically significant (*p* < 0.0001) on both terms, indicating a stable and reproducible phenotypic divergence between the two clones. Because plaque cloning was performed after multiple passages in Vero cells, the observed divergence may reflect the selection of pre-existing variants within the viral quasispecies or adaptation acquired during in vitro cell culture.

To identify the genetic changes potentially responsible for the observed phenotypic differences, we performed whole-genome sequencing of both viral variants. After the third passage, the viral RNA was isolated from the virus-containing supernatant and Illumina sequencing was carried out. Bioinformatic analysis revealed that the major distinguishing feature of the Delta s.p. variant was a 12-nucleotide insertion in the N-terminal domain of the spike glycoprotein, which occurs at high frequency and was the most plausible driver of the altered plaque morphology. This insertion, which was also confirmed by Sanger sequencing, consists of 12 nucleotides (Table 1) and has not been previously described (verified using NCBI blastn online tool (Supplementary Files, Figure S1, URL: https://github.com/ErmolaevaAElena/INSERTION-IN-THE-N-TERMINAL-DOMAIN-OF-THE-SARS-CoV-2-SPIKE-GLYCOPROTEIN.git, accessed on 20 December 2025)), disrupts the original reading frame, and introduces five novel amino acids (KRTRS).

Comparative analysis of spike protein sequences revealed the presence of Delta-associated substitutions L452R, T478K, and D950N relative to the Wuhan-Hu-1 reference strain (Table 1, Table S1). In addition, a deletion encompassing residues 679–685 (NSPRRAR) was identified, resulting in the loss of the S1/S2 furin cleavage site and the absence of the P681R substitution. This deletion is consistent with adaptation of Delta lineage–derived SARS-CoV-2 variants to propagation in Vero cells [32].

All functional experiments were performed using viral variant stocks after the fourth passage in Vero cells that did not contain further mutations beyond those described above.

Additional changes arising during Vero cell passaging were analyzed by NGS. During later passaging of the Delta s.p. variant, a V588L substitution in the RNA-dependent RNA polymerase (RdRp) emerged, appearing at passage 5 (34%) and increasing to 46% at passage 6. These mutations did not affect the results presented in this study.

**Figure 2:**
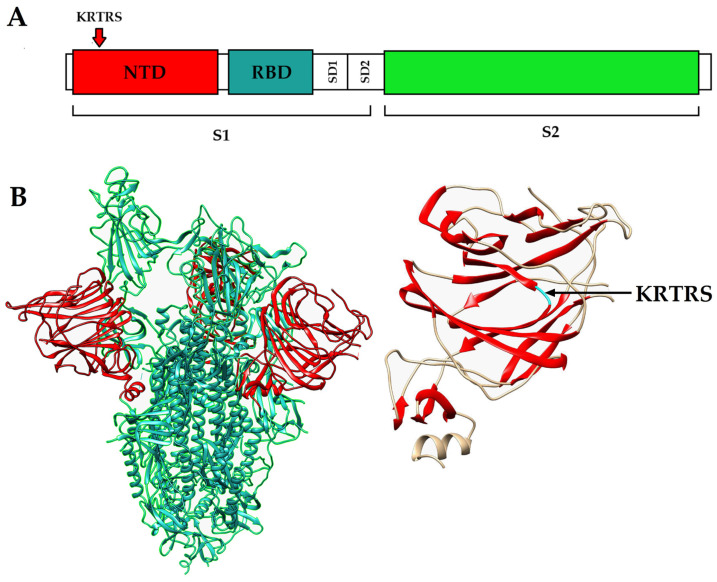
(**A**) Linearized region of the SARS-CoV-2 genome encoding the spike glycoprotein. The spike glycoprotein is divided into two subunits (S1 and S2). The S1 subunit contains two subdomains (SD1 and SD2), as well as a receptor-binding domain (RBD) and an N-terminal domain (NTD). The location of the KRTRS insertion is marked with an arrow in the NTD region of the spike glycoprotein in the Delta s.p. variant of the virus; (**B**) overall 3D structure of the trimer, whose monomers are spike glycoproteins. The 3 NTD regions of each monomer are marked in red. The NTD of one of the monomers is shown separately, with the KRTRS insertion region in the Delta s.p. variant marked in cyan. The RCSB Protein Data Bank IDs for the SARS-CoV-2 spike protein structures is 6ZGG, the visualization was made using UCSF chimera—1.19.

To visualize the structural context of the identified insertion, we examined its location within the domain architecture of the SARS-CoV-2 spike glycoprotein. The mutation occurred within the NTD of the S1 subunit, in a surface-exposed region that forms part of the β-sheet arrangement between the β6 and β7 strands (Figure 2). Two additional nonsynonymous substitutions in ORF1ab were detected in Delta l.p. variant at low abundance (~5%) in deep-sequencing data, indicating that they represent minor variants unlikely to account for the phenotypic divergence.

### 3.2. Phenotype-Dependent Differences in Spike Antibody Recognition

**Figure 3:**
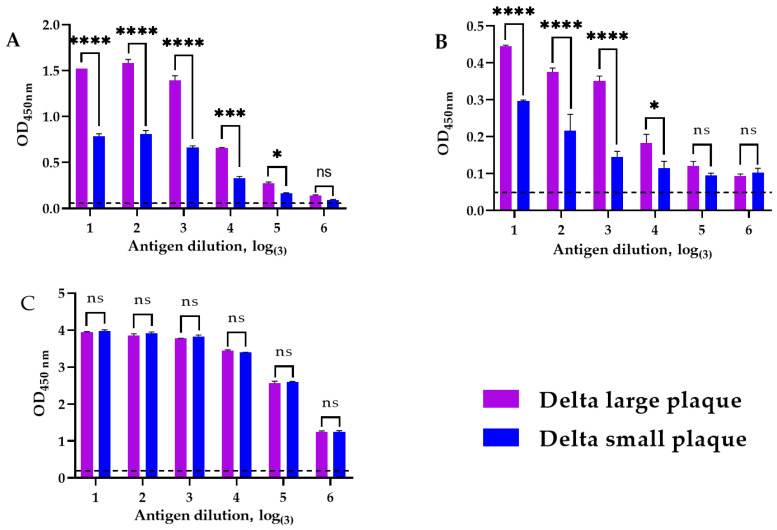
ELISA with: (**A**) polyclonal antibodies to the spike glycoprotein of SARS-CoV-2 virus; (**B**) monoclonal recombinant antibodies to the spike glycoprotein of SARS-CoV-2 virus; (**C**) polyclonal antibodies to the N (nucleocapsid) protein of SARS-CoV-2 virus. The antigen was subjected to titration by threefold serial dilution, beginning with the stock concentration. Dilution factors are presented as logarithms to the base 3 (log_3_). The dotted line indicates the background signal determined from wells without virus coating (negative control). All data are presented as averages with standard deviation * *p* < 0.05, *** *p* < 0.001, **** *p* < 0.0001 according to the multiple comparisons for two-way ANOVA using GraphPad Prism 8.2.1. Columns in the chart with a “ns” indicate that the observed differences between the variables are not statistically significant.

To evaluate the antigenic properties of the isolated SARS-CoV-2 variants, an enzyme-linked immunosorbent assay (ELISA) was performed. Viral preparations of the Delta s.p. and Delta l.p. variants (10^4^ TCID_50_/mL) were used as coating antigens and applied in triplicate to high-binding microplates for overnight adsorption. Binding of polyclonal and monoclonal anti-spike antibodies to the immobilized viral antigens was assessed, and antibody–antigen complexes were detected using HRP-conjugated secondary antibodies by measuring optical density at 450 nm (Figure 3).

ELISA analysis showed lower binding of both polyclonal and monoclonal anti-spike antibodies to the Delta s.p. variant relative to the Delta l.p. variant, reflected by reduced OD_450_ values across several antigen dilutions (Figure 3A,B). No significant differences were detected in the binding of polyclonal anti-N protein antibodies between the two variants (Figure 3C). One possible explanation for the reduced binding of anti-spike antibodies to the Delta s.p. variant is the presence of subtle structural differences in the spike protein that may influence epitope accessibility. Importantly, the comparable binding of anti-N protein antibodies suggests that the observed differences are specific to spike rather than reflecting differences in antigen loading.

### 3.3. Pathogenic Properties in Syrian Hamster Animal Model

**Figure 4:**
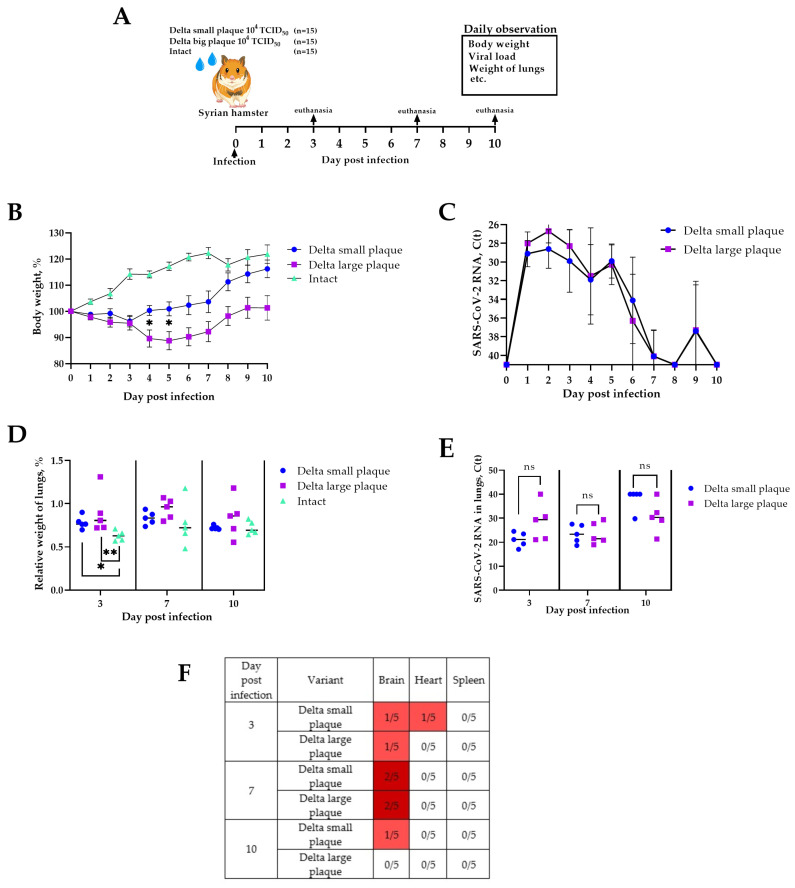
Pathogenic properties of the Delta l.p. and Delta s.p. viruses in Syrian hamster model: (**A**) experimental scheme; (**B**) weight dynamics post infection; (**C**) viral RNA load in oropharyngeal swabs post infection; (**D**) relative weight of lungs on days 3, 7, and 10 post infection; (**E**) viral RNA load in lungs. The Data in (**B**,**C**) are presented as averages with standard deviation; (**F**) detection of viral RNA in organs. The data (**D**,**E**) were analyzed using a nonparametric Mann–Whitney test for each day using GraphPad Prism 8.2.1. The data (**B**) according to the multiple comparisons for two-way ANOVA using GraphPad Prism 8.2.1; * *p* < 0.05, ** *p* < 0.001, no signature or “ns” indicate that the observed differences between the variables are not statistically significant.

Syrian hamsters were used as a non-lethal animal model to assess the pathogenic properties of the Delta SARS-CoV-2 variants upon intranasal infection. The study included three groups of 15 animals each: the first group was infected with 10^4^ TCID_50_ Delta s.p. variant (small-plaque phenotype), and the second group was infected with 10^4^ TCID_50_ Delta l.p. variant (large-plaque phenotype), and the third included uninfected intact animals (Figure 4A).

Daily monitoring included measuring body weight and clinical observations of animals. Infected animals of both groups started to lose weight on day one p.i. (Figure 4B). Statistically significant weight loss was observed from day four of the experiment. Animals infected with the Delta l.p. variant showed the most decrease in weight (Figure 4B). Animals infected with Delta s.p. started to gain weight on day four p.i., whereas the ones infected with Delta l.p. on day seven p.i. Therefore, animals infected with Delta s.p. recovered faster than animals infected with Delta l.p.

In an in vivo experiment, there was no statistically significant difference in viral load in oropharyngeal swabs between the Delta s.p. and Delta l.p. infected groups, according to the multiple comparisons for two-way ANOVA using GraphPad Prism 8.2.1 (Figure 4C). The lung edema was measured as relative weight of the lungs on days three, five, and seven p.i. upon euthanasia. There were statistically significant signs of edema in lungs of both infected groups only on day three p.i. (Figure 4D). However, we did not observe significant difference between infected groups in viral RNA load in the lungs on any term post infection (Figure 4E). Viral RNA was detected in the brain of animals infected with both Delta s.p. and Delta l.p. variants, with no statistically significant differences between the variants; detection in the heart was low, and viral RNA was not detected in the spleen (Figure 4F).

The gross pathology of infected and non-infected lungs was similar. During macroscopic examination, infected animals did not show significant differences in lung and pleural morphology from intact hamsters. Intact animals showed no signs of specific lung tissue damage at the macroscopic level. In lung surface of animals infected with Delta s.p. or Delta l.p. variants, no striking anatomical changes were detected including no pleural adhesions. The lungs of some animals from all three groups showed uneven coloration, which can be explained as an artifact of euthanasia.

**Figure 5:**
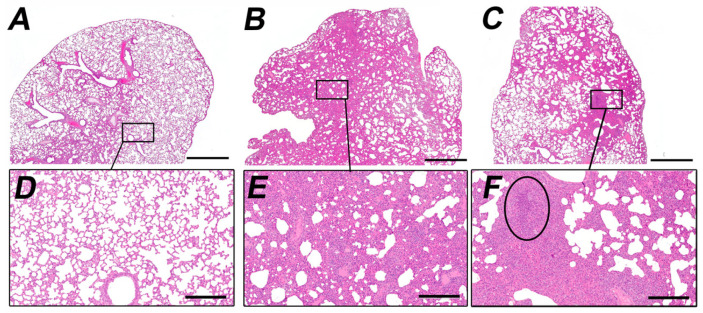
Changes in the lungs of experimental animals 7 d.p.i infected with Delta l.p. variant (**B**,**E**) and Delta s.p. variant (**C**,**F**). (**A**,**D**)—lung of an intact animal. Circle at (**F**) indicates focus of neutrophilic infiltration. Haematoxylin and eosin staining, magnification 20× (upper panel, (**A**–**C**)), 100× (lower panel, (**D**–**F**)). Scale bar 1 mm at (**A**–**C**) and 300 mkm at (**D**–**F**).

Pathological microscopic changes in the lungs of the infected animals of both groups corresponded to the initial stage of viral infection on day three p.i., medial stage of pneumonia at 7 d p.i. and late pneumonia stages at 10 d p.i.. Pneumonia in all animals in both experimental groups was at the same stage of morphological development at 3, 7, 10 d p.i. The most pronounced pneumonia was found at 7 d p.i. (Figure 5). The lung condition in the group infected with Delta l.p. (Figure 5B,E) at day seven post infection was insignificantly better than in the group infected with the insertion variant (Figure 5C,F). In the group of animals infected with the insertion variant, neutrophil infiltration was in some cases more pronounced, which indicates a slightly more severe pneumonia in animals of this group (Figure 5F).

**Figure 6:**
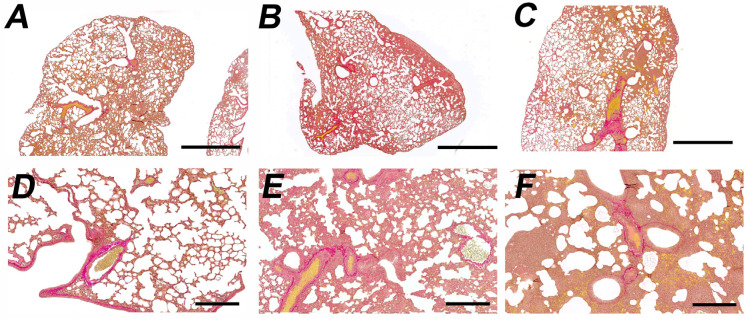
Collagen in the lungs of experimental animals 7 d.p.i infected with Delta l.p. variant (**B**,**E**) and Delta s.p. variant (**C**,**F**). (**A**,**D**)—lung of an intact animal. Pinkish red fibrils represent collagen. In both infected and intact lungs, collagen is organized as thick bundles surrounding bronchioles and blood vessels. No collagen accumulation is observed in areas of pneumonia (characterized by loss of the alveolar architecture) in animals infected with any of the viral variants. Fibrosis is not observed. Van Gieson’s trichrome stain, magnification 20× (upper panel, (**A**–**C**)), 100× (lower panel, (**D**–**F**)). Scale bar 2 mm at (**A**–**C**) and 300 mkm at (**D**–**F**).

No fibrotic changes were found in lung parenchyma of any group. Additionally, lung sections from three animals per experimental group (nine lungs in total) were stained with Van Gieson’s trichrome stain (Biovitrum, Russia); this analysis did not reveal evidence of collagen accumulation in lung parenchyma indicative of fibrosis (Figure 6). The severity of changes in lung tissue was similar in both variants of infection (Delta l.p and Delta s.p).

## 4. Discussion

Mutations may appear at each replication cycle of the SARS-CoV-2 genome due to RNA-dependent RNA polymerase errors [33]. Emerging mutations can affect transmissibility, such as the D614G mutation [34], and there is already evidence of mutations in the spike protein affecting its antigenicity and its neutralization by antibodies and consequently escape from acquired immunity [35]. Most mutations in NTD occur at sites accessible for binding; in addition, antibodies of the adaptive immune response of COVID-19 reinfected individuals bind to several sites of NTD [18]. Recent studies further indicate that mutations in the NTD can allosterically modulate spike protein functions, including S1/S2 cleavage, viral entry, and cell–cell fusion, in a variant- and context-dependent manner [36]. Therefore, it is important to study mutations in rapidly emerging coronavirus variants and their effect on the phenotypic properties of the virus, especially in the region encoding the NTD.

During our study, two phenotypically different variants of the coronavirus were isolated and characterized from the Delta SARS-CoV-2 isolate obtained from a patient, and the influence of genetic changes in the NTD of the spike glycoprotein on its phenotype were demonstrated. We discovered a previously undescribed 12-nucleotide insertion in the genome region encoding the NTD of the spike glycoprotein of the Delta s.p. variant (variant with small negative colonies) (Table 1). Importantly, this insertion was associated with a noticeable decrease in antibody binding in an in vitro assay, as evidenced by significantly lower optical density in ELISA using both polyclonal and monoclonal antibodies (Figure 3). This suggests that the 5-amino acid insertion in the NTD, located between the 6th and 7th β-strands, may alter the conformation of key antigenic sites in the domain, for example, because it can displace the N3 loop (which belongs to the NTD supersite [37]) or alter the bonds in the supersite, contributing to partial evasion of the immune response. Interestingly, this potential advantage did not lead to increased pathogenicity. Contrary to expectations, the Delta l.p. variant (without the insertion) caused more pronounced weight loss in the Syrian hamster model (Figure 4). Our results suggest that not all mutations that promote antibody evasion confer an advantage in vivo, which is an important factor in predicting the evolutionary development of SARS-CoV-2. Since we assume that the addition of five new amino acids changes the conformation of the spike protein, we can also assume a decrease in affinity for the ACE2 receptor. The effect of such mutations might be compensated by the epistatic effect of specific mutations [38]. In our case, we can assume that due to the small number of passages and replication cycles, compensatory mutations either did not appear or did not become established and are present in the population below the 5% threshold.

The in vivo experiment resulted in a statistically significant difference in weight loss of infected animals, but the difference in histological examination of pathological changes in lungs was not significant. This result may be related to the dose of virus used for intranasal administration, 10^4^ TCID [39]. Future investigations using higher infectious doses may help to better clarify the contribution of viral load to disease severity and to characterize the immune responses elicited by the Delta s.p. and Delta l.p. variants. This work is crucial for informing future vaccine strategies and therapeutic interventions against SARS-CoV-2 and its variants.

Both variants also contain additional mutations, two of which are variant-specific. However, the mutation in the Nsp1 region of the Delta l.p. variant was detected in only ~5% of viral genomes and is therefore unlikely to be phenotypically determinative. Likewise, a known mutation in the RdRp of the Delta s.p. variant is not expected to significantly affect neutralizing antibody responses (see Section 3.1).

As we advance our understanding of the immune response to SARS-CoV-2 variants, it is crucial to consider not only the mutations in variants but also how these changes can influence vaccine efficacy and public health strategies. For instance, the emergence of variants such as Delta (B.1.617.2) has demonstrated an enhanced ability to evade neutralizing antibodies, necessitating the confirmation of the effectiveness of current vaccines to ensure they remain effective against circulating strains [40]. Moreover, genomic control is essential to identify new mutations that may arise, particularly those located within the N-terminal domain (NTD) which have shown significant structural flexibility and potential for immune escape [19]. Future research should focus on integrating this knowledge into adaptive vaccine designs that can respond dynamically to viral evolution, such as incorporating multivalent vaccines targeting multiple epitopes across different variants, thereby improving overall efficacy against a broader range of circulating strains.

## Figures and Tables

**Table 1:** Sequencing of two SARS-CoV-2 virus variants with different plaque phenotypes.

Position	Reference (Wuhan Hu-1, NC_045512.2)	Delta Small Plaque	Delta Large Plaque	Amino Acid, Position	Spike Protein Domain
21,618	C	G	G	T19R	NTD
21,859–21,870	-	AAGATGGCGGAG	-	N99K, Ins(RTRS)	NTD
21,987	G	A	A	G142D	NTD
22,029–22,034	AGTTCA	Del (6)	Del (6)	Δ157–158	
22,917	T	G	G	L452R	RBD
22,995	C	A	A	T478K	RBD
23,403	A	G	G	D614G	SD2
23,596	TAATTCTCCTCGGCGGGCACG	Del (21)	Del (21)	Δ679–685 NSPRRAR	S1/S2
24,410	G	A	A	D950N	S2

## Data Availability

The original contributions presented in this study are included in the article/Supplementary Materials. Further inquiries can be directed to the corresponding authors.

## Supplementary Materials

The following supporting information can be downloaded at: https://www.mdpi.com/article/10.3390/v18030277/s1, Figure S1: Alignment of SARS-CoV-2 nucleotide sequences from 21,821 to 21,883 nucleotides; Table S1: Mutations contained in the Spike glycoprotein coding region in SARS-CoV-2 variants used to construct the alignment in Figure S1.
