# Supplementary material for: Insertion in the N-Terminal Domain of the SARS-CoV-2 Spike Glycoprotein Affects Antibody Recognition and Phenotypic Properties
Source: Viruses. 2026 Feb 24;18(3):277. doi: 10.3390/v18030277 (PMC13030607; doi:10.3390/v18030277)
Supplement: Supplementary file 1 [file viruses-18-00277-s001.zip › viruses-4094233-supplementary.pdf]

Article

INSERTION IN THE N-TERMINAL DOMAIN OF THE SARS-COV-2 SPIKE GLYCOPROTEIN AFFECTS ANTIBODY RECOGNITION AND PHENOTYPIC PROPERTIES

Elena A. Ermolaeva, Anna N. Zyrina, Dina I. Sirazova, Alexander S. Lunin, Anton S. Motov, Anastasia D. Chernavtseva, Olga S. Gancharova, Liubov I. Kozlovskaya, Anna A. Shishova, Alexandra A. Siniugina, Aydar A. Ishmukhametov

Supplementary materials

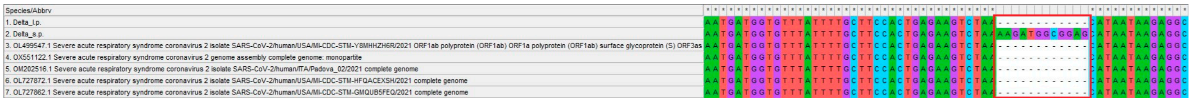

Figure S1. Alignment of SARS-CoV-2 nucleotide sequences from 21821 to 21883 nucleotides. The region containing the previously undescribed insertion is highlighted in red. Sequences for alignment were selected using NCBI blastn. The search was performed on the full genome sequence of the Delta s.p. variant, and the sequences with the highest identity (Per. Identity from 99.83% to 99.85%) were selected. The alignment file is available in the open Github repository (URL: https://github.com/ErmolaevaAElena/INSERTION-IN-THE-N-TERMINAL-DOMAIN-OF-THE-SARS-COV-2-SPIKE-GLYCOPROTEIN.git)

Table S1. Mutations contained in the Spike glycoprotein coding region in SARS-CoV-2 variants used to construct the alignment in Figure S1. The Wuhan-Hu-1 sequence was used as the reference genome.

Name	Mutation	Name	Mutation
Delta_lp_spike_CDS	T19R	Delta_sp_spike_CDS	T19R
Delta_lp_spike_CDS	G142D	Delta_sp_spike_CDS	N99K, Ins(RTRS)
Delta_lp_spike_CDS	E156G	Delta_sp_spike_CDS	G142D
Delta_lp_spike_CDS	L452R	Delta_sp_spike_CDS	E156G
Delta_lp_spike_CDS	T478K	Delta_sp_spike_CDS	L452R
Delta_lp_spike_CDS	D614G	Delta_sp_spike_CDS	T478K
Delta_lp_spike_CDS	D950N	Delta_sp_spike_CDS	D614G

OL499547_1:21563-25363_OL499547.1:21563-25363_...	T19R	Delta_sp_spike_CDS	D950N
OL499547_1:21563-25363_OL499547.1:21563-25363_...	G142D	OL727862_1:21559-25368_OL727862.1:21559-25368_...	T19R
OL499547_1:21563-25363_OL499547.1:21563-25363_...	E156G	OL727862_1:21559-25368_OL727862.1:21559-25368_...	G142D
OL499547_1:21563-25363_OL499547.1:21563-25363_...	P272T	OL727862_1:21559-25368_OL727862.1:21559-25368_...	E156G
OL499547_1:21563-25363_OL499547.1:21563-25363_...	L452R	OL727862_1:21559-25368_OL727862.1:21559-25368_...	L452R
OL499547_1:21563-25363_OL499547.1:21563-25363_...	T478K	OL727862_1:21559-25368_OL727862.1:21559-25368_...	T478K
OL499547_1:21563-25363_OL499547.1:21563-25363_...	D614G	OL727862_1:21559-25368_OL727862.1:21559-25368_...	D614G
OL499547_1:21563-25363_OL499547.1:21563-25363_...	Q675S	OL727862_1:21559-25368_OL727862.1:21559-25368_...	N679R
OL499547_1:21563-25363_OL499547.1:21563-25363_...	T676R	OL727862_1:21559-25368_OL727862.1:21559-25368_...	D950N
OL499547_1:21563-25363_OL499547.1:21563-25363_...	Q677R	OL727862_1:21559-25368_OL727862.1:21559-25368_...	E1262V
OL499547_1:21563-25363_OL499547.1:21563-25363_...	T678R	OM202516_1:21559-25359_OM202516.1:21559-25359_...	T478K
OL499547_1:21563-25363_OL499547.1:21563-25363_...	D950N	OM202516_1:21559-25359_OM202516.1:21559-25359_...	D614G

OM202516_1:21559-25359_OM202516.1:21559-25359_...	T19R	OM202516_1:21559-25359_OM202516.1:21559-25359_...	Q675S
OM202516_1:21559-25359_OM202516.1:21559-25359_...	G142D	OM202516_1:21559-25359_OM202516.1:21559-25359_...	T676R
OM202516_1:21559-25359_OM202516.1:21559-25359_...	E156G	OM202516_1:21559-25359_OM202516.1:21559-25359_...	Q677R
OM202516_1:21559-25359_OM202516.1:21559-25359_...	P251L	OM202516_1:21559-25359_OM202516.1:21559-25359_...	T678R
OM202516_1:21559-25359_OM202516.1:21559-25359_...	L452R	OM202516_1:21559-25359_OM202516.1:21559-25359_...	D950N
